# A global review of national guidelines of post‐exposure prophylaxis for the prevention of HIV

**DOI:** 10.1002/jia2.26333

**Published:** 2025-01-23

**Authors:** Marcus Maisano, Daniel Tran, Virginia Macdonald, Rachel C. Baggaley, Nathan Ford, Cheryl C. Johnson, Ying Zhang, Jason J. Ong

**Affiliations:** ^1^ Melbourne Sexual Health Centre Alfred Health Melbourne Victoria Australia; ^2^ School of Translational Medicine Faculty of Medicine Nursing and Health Sciences Monash University Melbourne Victoria Australia; ^3^ Melbourne Medical School The University of Melbourne Melbourne Victoria Australia; ^4^ Global HIV Hepatitis and STI Programmes World Health Organization Geneva Switzerland; ^5^ Faculty of Infectious and Tropical Diseases London School of Hygiene and Tropical Medicine London UK

**Keywords:** HIV, national guidelines, PEP, post‐exposure prophylaxis, prevention, review

## Abstract

**Introduction:**

The World Health Organization (WHO) recommends the use of antiretroviral drugs as post‐exposure prophylaxis (PEP) for preventing HIV acquisition for occupational and non‐occupational exposures. To inform the development of global WHO recommendations on PEP, we reviewed national guidelines of PEP for their recommendations.

**Methods:**

Policies addressing PEP from 38 WHO HIV priority countries were obtained by searching governmental and non‐governmental websites and consulting country and regional experts; these countries were selected based on HIV burden, new HIV acquisitions and the number of HIV‐associated deaths. We reviewed national guidelines to collate data on where PEP can be offered, who can prescribe PEP, PEP eligibility, recommended drug regime, linkage to other interventions, recommended investigations prescribed with PEP, HIV self‐test recommendation related to PEP and stopping rules for PEP.

**Results:**

In total, 46 guidelines from January 2010 to May 2023 across 36 countries were included, with 70% of documents published during or after 2020. There was significant variation across national guidelines regarding where PEP can be accessed and who can provide or prescribe PEP. Six countries (17%) described being able to access PEP from a primary care facility, four countries (11%) from hospitals and two (6%) from community‐based services. Only three countries (8%) specifically considered dispensing PEP by professionals other than doctors (e.g. nurses). None mentioned pharmacists as prescribers. We found a lack of consistency across countries regarding who is eligible for PEP, regimens used, interventions integrated into PEP provision and recommended investigations for PEP users. No country guidance provided considerations on using HIV self‐tests for starting or stopping PEP.

**Discussion:**

Despite PEP being recommended for more than three decades, many national policies were lacking in terms of PEP guidance. There are opportunities for countries to update and optimize guidance to consider ways to improve the accessibility of PEP. Greater efforts are needed to support the development of global consensus on how best to implement and integrate PEP, as well as how to include decentralization and task‐sharing to achieve sufficient scale for impact.

**Conclusions:**

Improving timely access to PEP and promoting PEP adherence could help contribute to reducing the incidence of HIV globally.

## 1 INTRODUCTION

HIV remains a major global public health issue, with an estimated 39.0 million (33.1–45.7 million) individuals living with HIV worldwide at the end of 2022. Although there are declines in HIV incidence in many countries in sub‐Saharan Africa, this is uneven, with some populations continuing to experience high incidence. In some countries, with predominantly key population epidemics, the rates of new HIV acquisitions continue to increase, and other countries report growing trends in new cases when previously on the decline [1]. In 2022, approximately 1.3 million (1.0–1.7 million) people were newly acquired with HIV [1]. To achieve the UN goal of ending HIV as a public health threat by the year 2030, it is imperative to improve current strategies to prevent new acquisitions.

In 2007, the World Health Organization (WHO) first recommended using antiretroviral drugs as post‐exposure prophylaxis (PEP) for preventing HIV acquisition to all types of exposure: occupational and non‐occupational, sexual or parenteral [2]. In the context of HIV, PEP refers to interventions provided aiming to prevent HIV acquisitions following potential exposure to HIV. PEP services have often included first aid, counselling (often including the assessment of the risk of exposure to the virus), HIV testing, and depending on the outcome of the exposure assessment, the prescription of a 28‐day course of antiretroviral drugs, with appropriate support and follow‐up [2]. It has also been stressed that PEP should be initiated as soon as possible after exposure, ideally within 72 hours [3]. Although randomized trials to assess the efficacy of PEP would be unethical, case‐control studies suggest that providing PEP within the first 72 hours after potential HIV exposure can prevent HIV acquisition [4, 5]. Early PEP using a single antiretroviral drug was prioritized for health workers following potential exposures in clinical settings [6]. Subsequently, PEP has been recommended for HIV prevention after sexual exposure and a combination of at least two antiretroviral drugs. WHO recommends PEP as part of a comprehensive HIV prevention strategy to reach the UN 2025 prevention targets of reducing the annual number of new HIV acquisitions to below 370,000. In addition to taking PEP after a potential exposure to HIV, other HIV prevention strategies include the use of pre‐exposure prophylaxis (PrEP) for at‐risk individuals.

While PEP is an important addition to the suite of HIV prevention options, uptake of this intervention remains low, particularly in low‐ and middle‐income countries (LMICs) and for key populations [7, 8]. Additionally, in many settings, PEP is unavailable outside medical facilities and tends to be used mainly in response to occupational exposure to HIV or following sexual assault.

To inform a WHO Global Guidelines Development Group for PEP (November 2023), we reviewed national guidelines to collate data on where PEP can be offered, who can prescribe PEP, PEP eligibility, recommended drug regimens and other interventions offered to PEP users.

## 2 METHODS

### 2.1 Selection of guidelines

This review follows the recommendations in the *Cochrane Handbook for Systematic Reviews* [9] and is reported according to the preferred reporting items for systematic reviews and meta‐analyses (PRISMA) guidelines [10]. We searched five electronic literature databases (Medline, EMBASE, Global Health, CINAHL and Web of Science) up to 16 May 2023. The search strategy was built around overarching terms, including “HIV,” “PEP,” “national,” “policy” AND “guideline,” and adapted to each database (Table S1). Studies published from January 2010 were included, and no language or geographical restrictions were placed on the search. However, the search did not return any policy documents, hence, we conducted our search on governmental and non‐governmental websites using keywords such as “HIV” and “PEP.” We also corresponded with WHO staff and country and regional experts to request for relevant documents. Data were collected between 16 May and 11 August 2023. No language and date restrictions were set. Eligible documents were related to HIV policy and published by the equivalent of the Ministry of Health in the respective country. Inclusion criteria required the documents to originate from one of the 38 WHO HIV priority countries, determined based on factors such as HIV burden, new HIV acquisitions and the number of HIV‐associated deaths (Table S2). The documents considered for inclusion were HIV antiretroviral treatment guidelines, HIV guidelines, specific PEP policies/guidelines, PrEP guidelines, as well as supplementary documents like pocket guides and fact sheets. Documents published in another language other than English were translated using Google Translate and cross‐checked by WHO staff where possible. Additionally, a supplementary manual search of references cited by the included documents was completed to locate relevant documents missed. References of the documents were manually created in Endnote reference management software (Clarivate, US). After removing duplicates, three researchers (YZ, MM and DT) independently screened the titles and content to identify potentially relevant documents. Each conducted a full‐text review according to the established inclusion and exclusion criteria, with a fourth reviewer (JO) resolving disagreements. No ethics approval was required as this is a review.

### 2.2 Data extraction

A data extraction spreadsheet was generated using Microsoft Excel. Pilot extraction was undertaken by three independent reviewers (YZ, MM and DT) on the first 10 documents to assess the appropriateness of the data extraction template. The template was modified as necessary to capture the relevant information. Data extraction was independently performed by the same three reviewers (YZ, MM and DT). Another reviewer (JO) resolved discrepancies. For each document, we extracted data regarding country, year of publication, type of document, country income level [11], world region [12], where PEP can be provided, who is eligible for PEP, screening for eligibility, who can prescribe PEP, minimum age for PEP, duration of PEP course, drug regime, linkage to other interventions, package of interventions prescribed with PEP, type of testing recommended, HIV self‐test recommendation, cost of PEP, stopping rules for PEP and inclusion of an HIV risk calculator. Documents containing no information about PEP or could not be translated were excluded from the review.

### 2.3 Data analysis

Descriptive statistics were used to summarize data. We conducted subgroup analyses according to the country income level and world region. The chi‐square or Fisher's exact test was performed to assess if there was any statistical association (*p* = 0.05) between the outcomes and country income level or world region.

## 3 RESULTS

**Figure 1:**
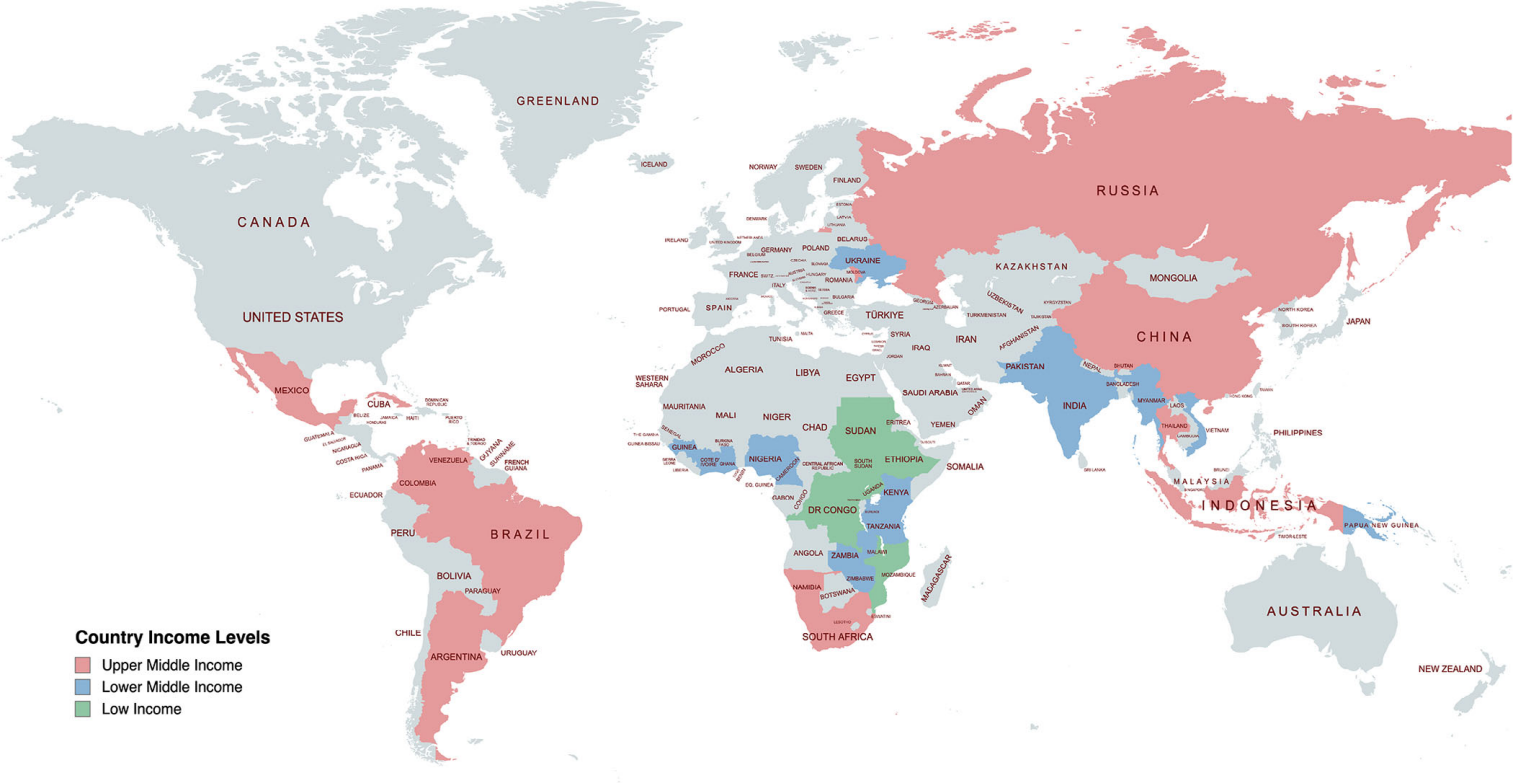
Included countries according to country income level (*N* = 36).

Following screening the 62 available policies from the 38 WHO HIV priority countries, 16 policies were excluded due to no relevant information on PEP being identified (Figure S1). As such, 46 policies across 36 countries were included (Table 1 and Figure 1). Publication dates of the 46 policies ranged from 2014 to 2023, with 70% of documents published on or after 2020. Most PEP‐related guidance was included as part of HIV treatment guidelines (46%), with a minority from PEP‐specific policy documents (12%). A complete list of guidelines reviewed is found in Table S3.

**Table 1:** Characteristics of policies included (*N* = 36)

	Number of countries (*N* = 36) *n* (%)
**Country income level** ^a^	
Upper middle	13 (36)
Lower middle	15 (42)
Low	8 (22)
**WHO regions**	
African region	19 (53)
Eastern Mediterranean region	1 (3)
European region	3 (8)
Region of the Americas	6 (17)
South‐East Asian region	4 (11)
Western Pacific region	3 (8)
**Language**	
English	19 (53)
Spanish	5 (14)
French	4 (11)
Portuguese	2 (6)
Chinese	1 (3)
Vietnamese	1 (3)
Russian	1 (3)
Romanian	1 (3)
Ukrainian	1 (3)
Indonesian	1 (3)

	**Number of policies (*N* = 46)** ***n* (%)**
**Year of publication**	
Unclear	1 (2)
2014	1 (2)
2016	1 (2)
2017	2 (4)
2018	2 (4)
2019	8 (17)
2020	5 (11)
2021	10 (22)
2022	14 (30)
2023	2 (4)

**Document type**	**Number of policy types (*N* = 65)** ***n* (%)**
HIV treatment guidelines	30 (46)
HIV guidelines	22 (34)
PEP guidelines	8 (12)
Other	5 (8)

^a^ Based on World Bank classification 2024.

### 3.1 PEP access

**Figure 2:**
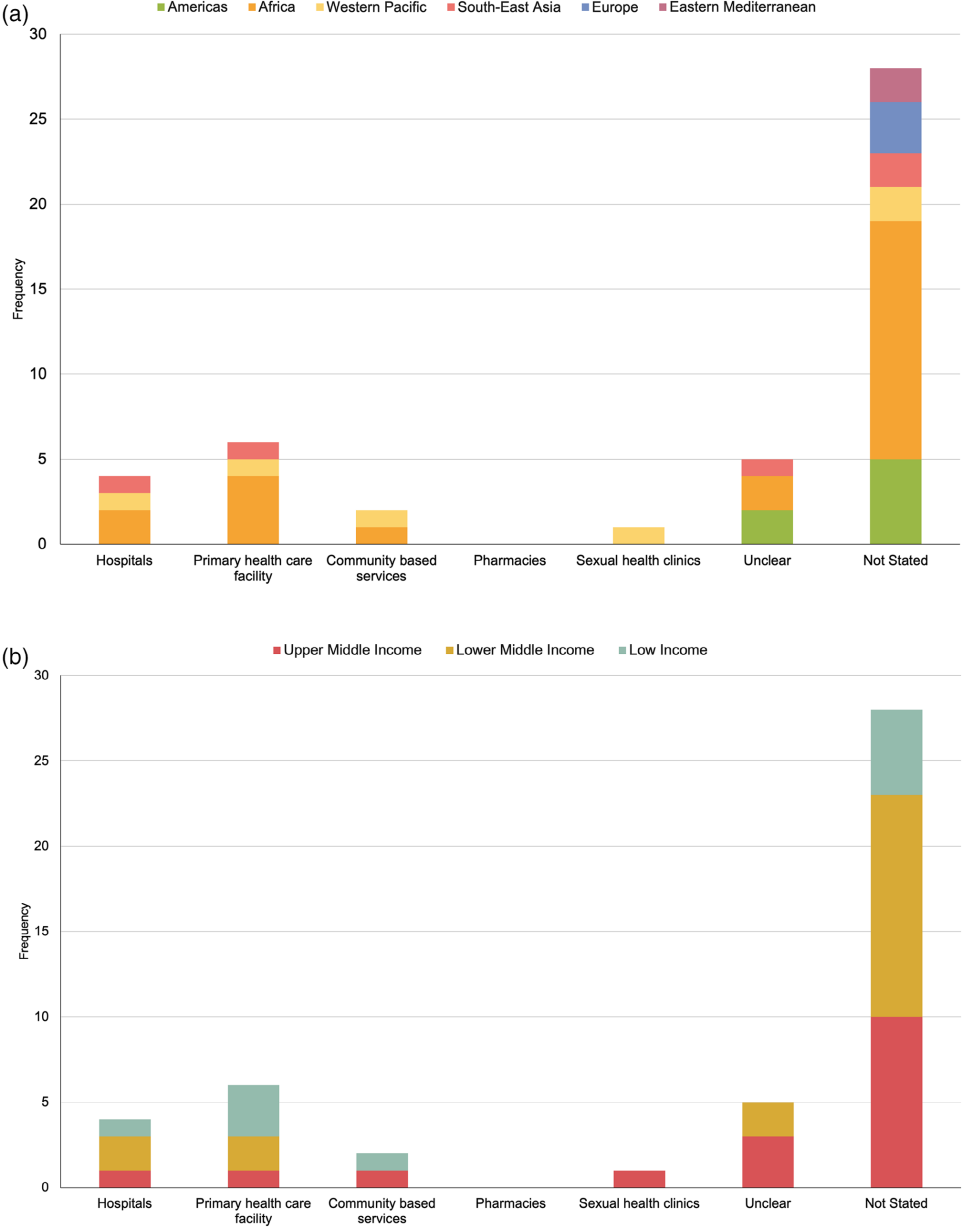
Location of PEP access, stratified by (a) regions of the world and (b) country income level (*N* = 46). Unclear = location of access to antiretrovirals was described in the document but not specifically related to PEP access (Brazil, Nigeria, Thailand and Zimbabwe).

PEP access (i.e. where PEP could be provided) was mentioned in various settings in guidelines from the African, South‐East Asian and Western Pacific regions. However, most countries (27/36, 75%) did not state where PEP could be accessed. Only six countries described being able to access PEP from a primary care facility (6/36, 16%), four countries from hospitals (China, Ethiopia, India and Nigeria) and two from community‐based services (China and Malawi). Figure 2 shows the classification of access locations by world region and country income level. We found no statistical significance between access locations and country income level (*p* = 0.9).

### 3.2 PEP eligibility

All countries except Guinea and Malawi mentioned using PEP for occupational exposure (34/36, 94%). All countries except for Guinea, Mozambique, Russia and Ukraine (32/36, 88%) mentioned prescribing PEP following an act of sexual violence. While Ethiopia, Guinea, Mozambique, Russia, Ukraine and Zambia did not mention using PEP following an episode of consensual sex, all other countries provided guidance for this setting (30/36, 83%). Fewer countries mentioned PEP for people who inject drugs (PWID) (21/36, 58%). Russia's guideline did allude to parenteral exposure; however, it was unclear whether this was intended for occupational exposure as well as for PWID. Other instances of non‐healthcare occupational parenteral exposure were mentioned by two countries (Columbia and Pakistan) which detailed its use for people who have received piercings and tattoos with non‐sterile equipment as well as for waste disposal workers. Other countries specifically mentioned PEP eligibility for people in custodial care (Nigeria), transgender persons (China, Indonesia and Nigeria) and pregnant mothers (Ghana and Democratic Republic of Congo).

Table 2 shows that most country guidelines (28/36, 77%) stated a minimum age of 0 for PEP. However, one country (Mexico) had a higher minimum age (2 years) but no specific justification was given.

**Table 2:** Minimum age and duration of PEP according to countries

		*n*/*N* (%)	Countries
Minimum age for PEP	No minimum age	28/36 (77%)	Brazil, Cameroon, China, Colombia, Cote D'Ivoire, Democratic Republic of Congo (DRC), Ethiopia, Ghana, India, Indonesia, Kenya, Malawi, Moldova, Myanmar, Namibia, Nigeria, Pakistan, Papua New Guinea, South Africa, South Sudan, Sudan, Thailand, Uganda, Ukraine, Venezuela, Vietnam, Zambia, Zimbabwe
	2 years old	1/36 (2%)	Mexico
	Not stated	4/36 (11%)	Cuba, Mozambique, Myanmar, Tanzania
	Unclear	6/36 (16%)	Argentina, Guinea, Nigeria, Russia, Rwanda, Ukraine
Duration of PEP	28 days	33/36 (91%)	Argentina, Brazil, Cuba, Cameroon, China, Colombia, Cote D'Ivoire, DRC, Ethiopia, Ghana, India, Indonesia, Kenya, Mexico, Moldova, Mozambique, Myanmar, Namibia, Nigeria, Pakistan, Papua New Guinea, Russia, Rwanda, South Africa, South Sudan, Sudan, Tanzania, Thailand, Uganda, Ukraine, Venezuela, Vietnam, Zambia
	28−30 days	1/36 (2%)	Indonesia
	30 days	3/36 (8%)	Colombia, Malawi, Zimbabwe
	Unclear	1/36 (2%)	Guinea

*Note*: Cameroon, China, Colombia, DRC, Indonesia, Malawi, Myanmar, Nigeria, Russia, Rwanda, South Africa, Ukraine, Vietnam and Zimbabwe had multiple documents reviewed and may appear more than once in the data series.

Screening tools were used to determine whether an individual is “eligible” for PEP in most countries (27/36, 75%). In addition, four countries (the Democratic Republic of Congo, Ethiopia, India and Kenya) provided risk calculators to quantify the probability of HIV transmission. For example, India's guideline provided a risk calculator that described HIV transmission through various routes: blood transfusion, perinatal transmission, sexual intercourse (stipulating anatomical sites), needle stick injury (occupational and non‐occupational) and mucous membrane exposure (including the oro‐nasal route). In contrast, the Democratic Republic of Congo's risk evaluation was described exclusively in terms of sexual practices.

### 3.3 Prescribers of PEP

When PEP prescribers were mentioned, several countries stated a medical doctor as the only practitioner able to prescribe (13/36, 36%). Cuba, Malawi and Zambia described registered nurses, specialist nurses trained in sexual health and advanced practice providers (including midwife technicians) as being able to prescribe PEP. Many countries did not mention who can prescribe PEP (66%, 24/36) and no country mentioned pharmacists as prescribers. We found no significant differences between prescriber information and country income level (*p* = 0.9).

### 3.4 Protocols for stopping PEP

Only three countries (Namibia, Tanzania and the Democratic Republic of the Congo) explicitly mentioned stopping PEP if a patient seroconverts during their PEP course. No policies specified any actions to take if there is further high‐risk exposure before finishing the PEP course, such as extending the course by 7 days or restarting the entire 28‐day course.

### 3.5 PEP drug regimes

All countries except Guinea (35/36, 97%) explicitly specified that PEP should be initiated within 72 hours after exposure to HIV. The majority recommended starting PEP as soon as possible (10/36, 28%), within 1−2 hours (5/36, 14%) or within 24 hours (2/36, 5%) post‐exposure. Guinea did not provide clear guidelines on the timing for PEP initiation.

Most countries specified a preferred 3‐drug PEP regime, which consisted of dual‐nucleoside reverse transcriptase inhibitor (NRTI) backbone agents with a third antiretroviral (ARV). All but three countries (Mexico, Mozambique and Guinea) included tenofovir disoproxil fumarate (TDF) as one of the NRTI agents. Mexico allocated TDF to be within the alternative regime and instead specified tenofovir alafenamide (TAF) or abacavir (ABC) within the preferred regime, with the latter to be used only if the patient has tested negative for HLA‐B*5701. Mozambique recommended using zidovudine (AZT) instead of TDF—but note that this guideline was from 2014. There was no information within the Guinean guidelines about choosing PEP drugs. Six guidelines among five countries included TAF as an option instead of TDF. Of these, one indicated that TAF was preferred over TDF (Mexico, 2021). One policy (China, 2021) indicated that TDF was still the preferred regime, with TAF being an alternative regime. The remaining guidelines stated that either drug could be used in the preferred regime—China (2020), Thailand (2020), Uganda (2022) and Zambia (2022). There was no clear pattern of TAF as an alternative among the world regions, while the proportion of countries recommending TAF was highest in the upper‐middle income countries group.

To complete the dual‐NRTI backbone, the guidelines specified lamivudine (3TC) (*n* = 17), emtricitabine (FTC) (*n* = 4) or either of these two drugs (*n* = 25). Among the 25 guidelines that stated either drug, a subgroup of seven policies stated that 3TC was preferred over FTC. Regarding country income levels, FTC but not 3TC was stated only by upper‐middle‐income countries, and a higher proportion of lower‐middle and low‐income level countries recommended 3TC compared to higher‐middle income level countries. Half of the guidelines from African countries indicated 3TC but not FTC as part of the dual‐NRTI backbone.

Table 3 shows the variety of the choice of third drug for the adult PEP regime. Most countries specified dolutegravir (DTG) as the third drug. A high proportion of African countries recommended protease inhibitors as a standalone option. On the other hand, a higher proportion of countries from the upper‐middle and lower‐middle income levels recommended DTG, and no upper‐middle income countries recommended using protease inhibitors only.

**Table 3:** Summary of the third drug recommended for PEP

Drug	Countries (*N* = 36)
**DTG**	Brazil, Côte d'Ivoire, Cuba, Democratic Republic of Congo, Kenya, Malawi, Namibia, Pakistan, Russia, South Africa, Tanzania, Vietnam, Zimbabwe
**DTG or**	China—RAL Ethiopia—EFV or LPV/r or ATV/r Ghana—LPV/r Mexico—BIC Sudan—EFV Ukraine—DRV/r or LPV/r or ATV/r or RAL
**DTG or (less preferred)**	Argentina—DRV/r Colombia—DRV/r India—LPV/r or EFV Indonesia—LPV/r or EFV Nigeria—EFV Papua New Guinea—LPV/r South Sudan—EFV or LPV/r or RAL or DRV/r Thailand—RPV or ATV/r or DRV/r Uganda—ATV/r or EFV Venezuela—RAL or ATV/r Vietnam—RAL or LPV/r Zambia—LPV/r or ATV/r
**Protease inhibitors**	Cameroon—LPV/r Moldova—LPV/r Mozambique—LPV Myanmar—LPV/r preferred, ATV/r or EFV less preferred Rwanda—ATV/r
**Others**	Nigeria—EFV
**2‐Drug option for low‐risk**	Ethiopia—TDF/3TC Mozambique—AZT/3TC
**No info**	Guinea

*Note*: Some countries may appear more than once due to multiple national guideline documents.

Abbreviations: 3TC, lamivudine; ATV/R, atazanavir/ritonavir; AZT, zidovudine; BIC, bictegravir; DRV/r, darunavir/ritonavir; DTG, dolutegravir; EFV, efavirenz; LPV/r, lopinavir/ritonavir; RAL, raltegravir; RPV, rilpivirine; TDF, tenofovir disoproxil fumarate.

Two countries (Ethiopia and Mozambique) stated a 2‐drug PEP regime option for low‐risk exposures among adults, alongside the usual 3‐drug regime for higher‐risk exposures. Low risk was defined by examples including “Minor mucocutaneous exposure to a small volume of blood for a short period (few seconds to minutes),” and high risk was defined by examples such as “large gauge punctured needle.”

More information about the recommended PEP regimens for children is summarized in Tables S4 and S5, demonstrating no consensus on the ideal regimen.

### 3.6 Interventions recommended with PEP provision

We found a range of recommended interventions alongside PEP, which varied across countries. The top four interventions included psychosocial support, sexually transmitted infection (STI) testing and/or prophylactic treatment, emergency contraception and PrEP (Table S6). Psychosocial support for survivors of gender‐based or sexual violence is recommended in 27 of 36 countries. STI testing and/or empirical treatment (i.e. given antibiotics pre‐emptively without waiting for STI testing results) are recommended in 27 countries for individuals who experienced sexual exposure. Commonly tested STIs include chlamydia, syphilis and gonorrhoea. In some countries, prophylactic treatment for STIs was recommended independently of the screening results. Except for China, India and Myanmar, all countries that provided STI testing also recommended emergency contraceptives for female victims of sexual assault. In addition, PrEP was recommended to be offered to individuals at continued risk of acquiring HIV and have tested HIV negative following completion of PEP (18/36, 50%). There was no further information on whether a seamless transition from PEP to PrEP without a gap in medications was recommended. None of the guidelines discussed the use of HIV self‐testing for use before starting and after stopping PEP.

Other PEP‐recommended interventions include harm reduction (support for sterile needles/syringes) for PWID and/or condom provision (16/36, 44%), hepatitis B/C testing and/or vaccination (11/36, 31%), tetanus toxoid for occupational exposure (healthcare workers, waste collectors) (6/36, 17%), criminal justice or legal interventions for post‐rape survivors (5/36, 14%), tuberculosis screening (3/36, 8%) and Covid screening (1/36, 3%). We found no statistically significant association between the interventions integrated with PEP and country income level (*p* = 0.471–0.763) or region of the world (*p* = 0.396–0.947).

Following initiation of PEP, some policies recommended that the individuals undergo a comprehensive set of serology tests, including HIV serology, full blood count, hepatitis B/C screening and vaccination (if no prior vaccination done), liver function test, renal function test, STI screening and pregnancy test at different intervals, which varied across the different countries (Table 4). No country guidance provided considerations on using HIV self‐tests for starting or stopping PEP.

**Table 4:** Summary of different tests performed at varying time points after PEP initiation

Type of test	Time point	*n*/*N* (%)	Countries
HIV	Week 0	34/36 (94%)	All except Guinea, Mexico
	Week 4–6	24/36 (67%)	Argentina, Brazil, Cameroon, China, Cote D'Ivoire, Cuba, Ethiopia, Ghana, India, Kenya, Mexico, Mozambique, Namibia, Nigeria, Pakistan, Russia, Rwanda, South Africa, Sudan, Thailand, Uganda, Venezuela, Vietnam, Zambia
	> Week 6	28/36 (78%)	Brazil, Cameroon, China, Colombia, Cote D'Ivoire, Cuba, India, Kenya, Malawi, Mexico, Moldova, Mozambique, Myanmar, Namibia, Nigeria, Papua New Guinea, Russia, Rwanda, South Africa, South Sudan, Tanzania, Thailand, Uganda, Ukraine, Venezuela, Viet Nam, Zambia, Zimbabwe
Hepatitis	Week 0​	19/36 (53%)	Argentina, Brazil, China, Colombia, Cote D'Ivoire, Cuba, DRC, India, Kenya, Myanmar, Namibia, Nigeria, Pakistan, South Africa, Tanzania, Thailand, Venezuela, Vietnam, Zimbabwe
	Week 4–6​	5/36 (14%)	Argentina, Brazil, Cote D'Ivoire, Cuba, Mozambique
	> Week 6	6/36 (17%)	Brazil, Colombia, Cote D'Ivoire, Cuba, Mozambique, Thailand
Full blood count	Week 0	8/36 (22%)	Cameroon, Colombia, Cote D'Ivoire, Cuba, India, Mozambique, Nigeria, Thailand
	Week 2	6/36 (17%)	Colombia, Cuba, India, Mozambique, Nigeria, Tanzania
	Week 4–6	4/36 (11%)	Colombia, Cuba, India, Mozambique
	> Week 6	1/36 (3%)	Cuba
Liver function test	Week 0	11/36 (31%)	Argentina, Cameroon, China, Colombia, Cote D'Ivoire, Cuba, India, Mozambique, Nigeria, Tanzania, Thailand
	Week 2	6/36 (17%)	China, Colombia, Cuba, Mozambique, Nigeria, Tanzania
	Week 4–6	5/36 (14%)	Argentina, China, Colombia, Cuba, Mozambique
	> Week 6	0	None
Renal function test	Week 0	10/36 (28%)	Argentina, Cameroon, Colombia, Cuba, Kenya, Namibia, Nigeria, Rwanda, Tanzania, Thailand
	Week 2	4/36 (11%)	Colombia, Cuba, Nigeria, Tanzania
	Week 4–6	4/36 (11%)	Argentina, Colombia, India, Rwanda
	> Week 6	2/36 (5%)	Cuba, Rwanda
Pregnancy test	Week 0	10/36 (28%)	Argentina, Brazil, Cameroon, Colombia, DRC, Kenya, Mexico, Papua New Guinea, Thailand, Venezuela
	Week 4–6	2/36 (6%)	Brazil, Thailand
	> Week 6	3/36 (8%)	Brazil, Papua New Guinea, Thailand
STI test	Week 0	22/36 (61%)	Argentina, Brazil, Cameroon, China, Colombia, Cuba, DRC, Ethiopia, Ghana, India, Indonesia, Malawi, Mexico, Myanmar, Namibia, Pakistan, Rwanda, South Africa, South Sudan, Thailand, Uganda, Viet Nam
	Week 4–6	4/36 (11%)	Argentina, Brazil, China, Thailand
	> Week 6	2/36 (6%)	Brazil, Thailand

Abbreviations: HIV, human immunodeficiency virus; STIs, sexually transmitted infections.

## 4 DISCUSSION

This study synthesizes data from PEP guidelines from countries selected based on HIV burden, new HIV acquisitions and HIV‐associated deaths. Although many policies did not provide details on who can provide PEP and where it can be delivered, in those that provided details, we found significant variations related to where PEP can be accessed and who can provide or prescribe PEP. There were also variations across countries on who is eligible for PEP, regimens used, interventions recommended with PEP provision and recommended investigations for PEP users. Our review highlights gaps and lack of clear guidance in national guidelines, which could improve the accessibility of PEP through decentralization and task shifting. Only a minority of guidelines specifically considered PEP access outside hospitals or dispensing PEP by professionals other than doctors.

Few guidelines mentioned accessing PEP in a location other than a tertiary medical centre (or hospital). A recent scoping review found that long distances, healthcare professional shortages, misinformation about PEP and stigmatization underpinned why the populations surveyed could not access and adhere to PEP [13]; the authors suggested that allowing trained pharmacists to dispense PEP could increase access. In South Africa, it has been suggested that the community pharmacy sector could improve the delivery of HIV services, including PEP in LMICs, where pharmacies are often the first point of contact with the healthcare system and pharmacists are the most consulted healthcare providers [14]. Since 2014, a Canadian emergency ward in Ontario has successfully implemented a nurse‐led PEP initiative. In their model of delivery, patients who felt they were unable to return to the HIV clinic would be eligible to visit a community outpatient pharmacy instead [15]. In the United States, as described by Scarnati and colleagues, since 2023, at least 13 states have successfully passed legislation for pharmacists to prescribe PEP [16]. A recent modelling study demonstrated the potential for making PEP widely and freely available without a doctor's prescription, along with a push towards community education about all aspects of HIV, which could be a cost‐effective strategy in many high‐burden settings in LMICs [17].

Although PEP has been recommended for decades, access and uptake have been limited, and there is an insufficient focus on optimizing PEP delivery. This is in contrast to the widespread delivery and high uptake of PrEP in many countries since its recommendation by the WHO in 2015 [18, 19]. There is a growing recognition that PEP delivery needs to be simplified and services decentralized. National guidelines should consider being more explicit in facilitating decentralized services and providing regulatory and legislative guidance for PEP to be delivered outside health facilities by other health workers and community workers. Nevertheless, there are newer models of PEP provision, such as self‐start PEP (PEPSE) or PEP‐in‐Pocket (PIP), that empower at‐risk individuals with more immediate access to PEP. PEPSE involves providing these individuals with a starter pack of PEP in advance, enabling them to initiate treatment immediately after a potential exposure. It ensures that individuals can begin treatment without the delay of seeking medical care post‐exposure, which is particularly beneficial in remote or underserved areas or during times outside regular healthcare hours or in individuals who hesitate to seek medical care due to fear of stigmatizing treatment from healthcare staff. Furthermore, individuals are empowered to take control over their health decisions, reducing reliance on healthcare providers for immediate response and potentially increasing adherence to the PEP regimen. A randomized open‐label study on PEPSE in the United Kingdom demonstrated that self‐start home PEPSE was safe to take and reduced the average time from potential exposure to HIV to the first PEPSE dose, from 29 to 7 hours [20]. Moreover, the self‐reported adherence to the PEPSE pack was 100% in all 43 participants [20]. Comparatively, PIP takes the concept of PEPSE further by providing high‐risk individuals with the full 28‐day course of PEP in advance. By having the entire course of medication on hand, individuals are ensured continuity of treatment from the moment of exposure through the full 28‐day regimen. This also reduces the burden on healthcare systems and individuals by minimizing follow‐up visits solely for prescription refills. Studies have demonstrated the acceptability and feasibility of PIP. A cohort study of 111 patients in Canada found that PIP was self‐initiated 69 times by 35 individuals, and no HIV seroconversions were identified [21]. Decentralizing the provision of PEP and innovative delivery methods may help to increase PEP access, allowing individuals to begin PEP as early as possible, even after potential exposure to HIV.

A wide range of ARV drug combinations were recommended. Many did reflect recent WHO recommendations for TDF + 3TC (or FTC) as the preferred backbone and DTG as the preferred third drug for HIV PEP, and when available. A third of the guidelines were published before 2020, and some PEP drug combinations recommended older drugs, which have more serious adverse events and less efficacy when used in treatment combinations, such as zidovudine with lamivudine [22], underscoring the importance of pre‐2020 guidelines to consider updates of their recommended PEP drugs. Also noteworthy was that only two countries specified the use of two‐drug regimens for low‐risk exposures, given that there is no evidence that three drugs are necessary for PEP [23] and only recommending three‐drug regimens for PEP could result in unnecessary costs for scaling up access to PEP. There were also some trends noted between certain drug recommendations and a country's income level, with higher‐income countries recommending TAF, a more costly drug compared to generic TDF, and DTG, a more costly drug compared to protease inhibitors [24]. However, it was beyond the scope of our study to evaluate how ARV recommendations were influenced by availability, cost, pharmaceutical industry presence in‐country or whether a country receives subsidized ARVs from PEPFAR.

Depending on the nature of the exposure, offering a range of additional interventions alongside PEP is essential. Individuals with occupational exposure (e.g. needlestick injuries) need targeted interventions, such as educational programmes on properly handling sharps equipment. Similarly, individuals exposed to HIV outside clinical settings require different interventions, including appropriate STI testing and/or treatment. Additionally, survivors of sexual violence require specialized psychosocial support, mental healthcare to manage the traumatic event's psychological repercussions and additional legal assistance if needed [25]. Some PEP users who may continue to have substantial HIV risk (e.g. men who have sex with men and PWID) could potentially benefit from transitioning to PrEP. However, as PEP is often prescribed by acute responders, such as practitioners in emergency departments, dedicated resources may be required to facilitate effective transitions for individuals who switch to PrEP to receive ongoing care in primary care or decentralized settings [23]. Interventions such as automated prompts in electronic health records or clinical decision aids to facilitate patient‐provider discussions could be implemented to prevent missed opportunities to transition to PrEP. Additionally, there is a continued need for clear clinical practice guidelines, as current guidelines often lack explicit recommendations for transitioning PEP users to PrEP, resulting in inconsistent practices across different healthcare settings. Often, primary care practitioners or advanced practice providers in decentralized settings may not be adequately trained to discuss PrEP or may lack up‐to‐date knowledge about its benefits and protocols, leading to patients completing the PEP regimen without being informed about PrEP as a longer‐term preventive measure. Including PrEP linkage in PEP guidelines improves continuity of care for individuals at high risk of HIV acquisition. This continuity is crucial for maintaining the momentum of prevention efforts initiated by PEP. By seamlessly transitioning patients to PrEP, healthcare providers can offer sustained protection against HIV, reducing the likelihood of seroconversion following repeated exposures.

Although offering a range of interventions for PEP users would be ideal, this may not be feasible in decentralized services and can be tailored to context. For example, in community‐based PEP delivery, these could include the provision of emergency contraceptives, STI testing and prophylactic STI treatment. Task‐sharing from specialist care to primary care may reduce delays in starting PEP. In addition to primary care physicians, pharmacists, nurses and community health workers at community‐based services can be trained to deliver and prescribe PEP to increase access, support the earlier start of PEP and potentially reduce costs. For instance, nurse‐led PrEP delivery is a viable approach for PrEP delivery that can increase PrEP service capacity without needing extra resources [26]. Alternatively, offering telehealth services (i.e. online consultation with a clinician followed by PEP, STI empirical treatment, emergency contraceptives, etc.) at pharmacies or community‐based services may increase access to PEP in the community.

Although PEP is available in many countries, there is a lack of consensus on the types of diagnostic tests performed with PEP. WHO recommends HIV testing using a self‐test or through a standard national testing algorithm before starting and following completion of PEP. This review noted that some countries recommend baseline liver and kidney function tests and subsequent re‐testing at 2 weeks or 1 month. In a recent study examining baseline renal and liver testing in 1051 patients using TDF + FTC + DTV for PEP without any known history of kidney and liver disease, they reported that these tests rarely resulted in changes in the PEP regimen (0.2%) [27]. As PEP is only taken for 28 days, there should be limited safety concerns, and there should be no need for routine renal and liver testing and monitoring for a healthy patient population taking PEP. In our review, we found that none of the national guidelines reviewed specifically mentioned HIV self‐test (HIVST) for use before starting and after stopping PEP. HIVST could be an effective addition to community delivery of PEP as HIVST offers a convenient and accessible means for individuals to monitor their HIV status, reducing the need for clinic visits after completing PEP [28]. A study conducted in Kenya reported the high acceptability of HIVST among healthcare workers and that they liked having access to HIVST, so they did not have to test in clinics where they worked or around their peers [29]. Given the informal usage of HIVST by healthcare workers to self‐test for HIV, there is potential for HIVST to be utilized by PEP users as well. Furthermore, individuals using self‐tests are empowered to actively participate in their healthcare decisions, promoting better adherence to PEP [30] and encouraging engagement in follow‐up testing [31]. By decentralizing follow‐up testing, healthcare systems can allocate resources more efficiently, focusing on individuals who need specialized care or support. Although implementing HIVST as part of PEP programmes has the potential to optimize the delivery of PEP services further, making them more client‐centred and accessible, it also requires careful planning, community education, counselling and quality control to ensure that individuals who self‐test receive the necessary support and linkage services.

Our review has several strengths. To ensure the robustness of our results, relevant data were triple‐extracted from the national guidelines. We included multiple countries and languages and validated our data extractions with WHO. These findings should be interpreted in light of the following limitations. First, we only included 36 countries, limiting the ability to assess statistical significance according to country‐income levels or world regions. Second, the guidelines were translated using Google Translate (except for Mexico and China). This may have the potential for inaccurate translations. Third, there may be additional key documents that countries may use for PEP provision that we may not have identified. Last, multiple guidelines were observed in nine countries; thus, ambiguity exists regarding which specific guidelines are adhered to by those countries in actual implementation.

Our review has some limitations. First, we did not investigate the use of patient‐centred language in our review because although where a translated version of a guideline was not available, we made our best effort to translate it, the style of language used may not be as patient‐centred and accurate as what could be understood by the clinician. Second, while we are aware that Cochrane is generally used when comparing randomized trials, we are comparing national policies here. Since our approach is not typical and without outcome data, there is no way to definitively determine which guideline is superior.

## 5 CONCLUSIONS

Our review of national guidelines for PEP found significant variations in how PEP is offered. There are opportunities for countries to update guidance to consider ways to improve the accessibility of PEP through decentralization beyond hospitals and task shifting to nurses and pharmacists, as well as using self‐tests. Improving timely access to PEP among those who need it would prevent HIV acquisitions following potential exposure to HIV, contribute to reducing the high incidence of HIV globally and achieve the UN goal of ending HIV as a public health threat by the year 2030.

## COMPETING INTERESTS

None of the authors has any competing interests to declare. The findings and conclusions in this report are those of the authors and do not necessarily represent the official position of the WHO.

## AUTHORS’ CONTRIBUTIONS

RCB, VM and JJO conceived the idea. MM, YZ and DT conducted the screening and extraction of data. JJO, MM, YZ and DT wrote the first draft of the manuscript. All authors contributed to the final manuscript and approved it for publication.

## FUNDING

JJO is supported by the Australian National Health and Medical Research Council (NHMRC) Emerging Leadership Investigator Grant (GNT1193955). YZ is supported by an Australian Government Research Training Programme (RTP) scholarship.

## Supporting information


**Table S1** Literature search strategy
**Table S2** List of priority countries selected by WHO
**Figure S1** PRSIMA flow diagram
**Table S3** Names of guidelines reviewed
**Table S4** Children – 2‐Drug Backbone
**Table S5** Children – Choice of 3^rd^ Drug
**Table S6** Interventions integrated with PEP (N = 36)

## Data Availability

Data will be made available upon request made to the corresponding author.
