# Supplementary material for: A global review of national guidelines of post‐exposure prophylaxis for the prevention of HIV
Source: J Int AIDS Soc. 2025 Jan 23;28(1):e26333. doi: 10.1002/jia2.26333 (PMC11755062; doi:10.1002/jia2.26333)
Supplement: Supplementary file 1 — Table S1 Literature search strategy Table S2 List of priority countries selected by WHO Figure S1 PRSIMA flow diagram Table S3 Names of guidelines reviewed Table S4 Children – 2‐Drug Backbone Table S5 Children – Choice of 3rd Drug Table S6 Interventions integrated with PEP (N = 36) [file JIA2-28-e26333-s001.docx]

**Supplementary**

Table of Contents

[Table S1 Literature search strategy 2](#_Toc171416361)

[Table S2 List of priority countries selected by WHO 3](#_Toc171416362)

[Figure S1 PRSIMA flow diagram 4](#_Toc171416363)

[Table S3 Names of guidelines reviewed 5](#_Toc171416364)

[Table S4 Children – 2-Drug Backbone 9](#_Toc171416365)

[Table S5 Children – Choice of 3^rd^ Drug 10](#_Toc171416366)

[Table S6 Interventions integrated with PEP (N= 36) 11](#_Toc171416367)

## Table S1 Literature search strategy

| **Search Number** | **Search strategy** |
| --- | --- |
| 1 | (“HIV infection*” or “ HIV” or “HIV1” or “HIV2” or “HIV type 1” or “HIV type 2” or “human immunodeficiency virus” or “human immun* adj3 deficiency virus” or “acquired immunodeficiency syndrome” or “acquired immun* adj3 deficiency syndrome” or “Sexually transmitted diseases, Viral”).mp |
| 2 | (“PEP” or “Post exposure prophylaxis” or “post-exposure prophylaxis”).mp |
| 3 | (“national” or “guideline*” or “polic*”).mp |
| 4 | 1 and 2 and 3 |
| 5 | Limit 4 to humans |

## Table S2 List of priority countries selected by WHO

| Angola | Myanmar |
| --- | --- |
| Argentina | Namibia |
| Brazil | Nigeria |
| Cameroon | Pakistan |
| China | Papua New Guinea |
| Colombia | Philippines |
| Cote D'Ivoire | Russia |
| Cuba | Rwanda |
| Democratic Republic of Congo | South Africa |
| Ethiopia | South Sudan |
| Ghana | Sudan |
| Guinea | Tanzania |
| India | Thailand |
| Indonesia | Uganda |
| Kenya | Ukraine |
| Malawi | Venezuela |
| Mexico | Vietnam |
| Moldova | Zambia |
| Mozambique | Zimbabwe |

## Figure S1 PRSIMA flow diagram


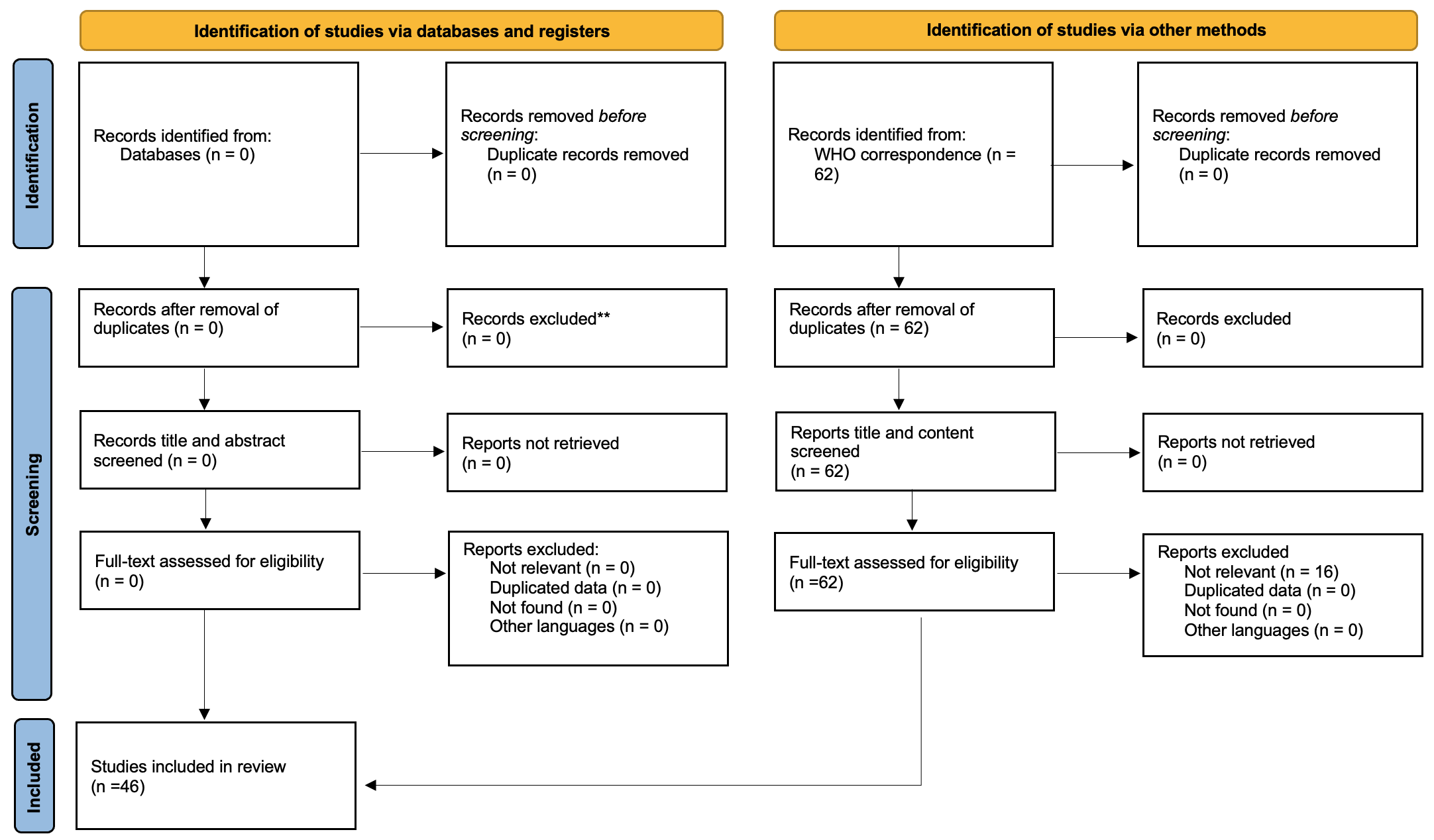


## Table S3 Names of guidelines reviewed

| **Country** | **Year** | **Title** | **Language** | **PEPFAR-supported?** |
| --- | --- | --- | --- | --- |
| Argentina | 2019 | Profilaxis Post Exposición para el VIH en personas adultas (Post Prophylaxis Exposure for HIV in adults) | Spanish |  |
| Brazil | 2021 | Profilaxia Pós-exposição (PEP) de Risco À Infecção pelo HIV, Ist e Hepatites Virais (Clinical Protocal and Guidelines Therapeutic for Prevention Post-exposure (PEP) of Risk to Infection from HIV and Viral Hepatitis) | Portuguese |  |
| Cameroon | 2023 | Directives Nationales de prise en charge du VIH au cameroun (National HIV Care Guidelines in Cameroon) | French | ✓ |
| Cameroon | 2023 | Directives de prevention et de prise en charge du VIH/SIDA au Cameroun (Guidelines for the prevention and care of HIV/AIDS in Cameroon) | French | ✓ |
| China | 2020 | 艾滋病病毒暴露后预防技术指南 (Guidelines for HIV post-exposure prophylaxis techniques) | Chinese |  |
| China | 2021 | 中国艾滋病诊疗指南（2021 年版）((Guidelines for Diagnosis and treatment of AIDS in China (2021 edition)) | Chinese |  |
| Colombia | 2017 | Protocolo para la atención por exposición de riesgo biológico laboral o no laboral, ante las infecciones de transmisión sexual, el Virus de Inmunodeficiencia Humana, el virus de la Hepatitis B y el virus de la Hepatitis C. (Protocol for care due to Occupational or Non-occupational biological risk exposure, before sexually transmitted infections, HIV & Hepatitis viruses) | Spanish |  |
| Colombia | 2022 | Actualización del protocolo de profilaxis post exposición a (Update of the post-exposure prophylaxis protocol) | Spanish |  |
| Cote d'ivoire | 2019 | Directives 2019 de prevention et de prise en charge des personnes vivant avec lev VIH en Côte D’Ivoire. (2019 guidelines for prevention and care of people living with hiv in Côte D'Ivoire) | French | ✓ |
| Cuba | 2019 | Plan Estratégico Nacional para la prevención y control de las ITS el VIH y las Hepatitis. (National Strategic Plan for the prevention and control of STIs , HIV and Hepatitis) | Spanish |  |
| Democratic Republic of Congo | 2021 | Guide pratique du prestataire du site de prise en charge de populations cles. (Practical guide from service providers for high load populations | French | ✓ |
| Democratic Republic of Congo | 2021 | Guide de prise en charge integree du VIH en Republique Democratique du Congo. (Guide to integrated management of HIV in Democratic Republic of Congo) | French | ✓ |
| Ethiopia | 2022 | National guidelines for comprehensive HIV prevention, care and treatment | English | ✓ |
| Ghana | 2022 | Consolidated guidelines for HIV care in Ghana | English | Yes, but regional programs |
| Guinea | - | Aperçu prise en charge VIH en Guinée (Overview HIV Care in Guinea) | French |  |
| India | 2021 | National Guidelines for HIV Care and Treatment 2021 | English |  |
| Indonesia | 2022 | Penanggulangan Human Immunodeficiency Virus, Acquired Immunodeficiency Syndrome, dan Infeksi Menular Seksual (Treatment of Human Immunodeficiency Virus, Acquired Immuno deficiency syndrome, and sexually transmitted infections) | Indonesian |  |
| Indonesia | 2019 | Pedoman Nasional pelayanan kedokteran tata laksana HIV (National guidelines for Medicine Services) | Indonesian |  |
| Kenya | 2022 | Kenya HIV Prevention and Treatment Guidelines, 2022 | English |  |
| Malawi | 2022 | Clinical Management of HIV in Children and Adults | English | ✓ |
| Mexico | 2022 | Guía de atención para otorgar profilaxis pre-exposición (Guidelines for antiretroviral management of people living with HIV) | Spanish |  |
| Moldova | 2018 | Profilaxia Post-expunere la infecția cu HIV Protocol clinic național (Post-exposure Prophylaxis for HIV infection National Clinical Protocol) | Romanian |  |
| Mozambique | 2014 | Directriz para integração dos serviços de prevenção, cuidados e tratamento do HIV e sida para a população chave no sector da saúde (Treatment Guide antiretroviral & Opportunist infections of adult, young pregnant and child) | Portuguese | ✓ |
| Myanmar | 2017 | Guidelines for the clinical management of HIV infection in Myanmar | English |  |
| Myanmar | 2018 | Amendment to the Guidelines for the clinical management of HIV infection in Myanmar 5th edition | English |  |
| Namibia | 2019 | National Guidelines for Antiretroviral Therapy Sixth edition, August 2019 | English | ✓ |
| Nigeria | 2020 | National Guidelines for HIV Prevention, Treatment and Care | English | ✓ |
| Nigeria | 2021 | Consolidated Service Delivery Guidelines on HIV and STIs for Key Populations in Nigeria | English | ✓ |
| Pakistan | 2022 | Consolidated Guidelines for the Prevention and Treatment of HIV and AIDS in Pakistan 2022 | English |  |
| Papua New Guinea | 2019 | Papua New Guinea national guidelines for HIV care and treatment | English | Yes, but regional programs |
| Russia | 2020 | ВИЧ-инфекция у взрослых (HIV Infection in Adults) | Russian |  |
| Rwanda | 2016 | National guidelines for prevention and management of HIV and stis | English | ✓ |
| South Africa | 2022 | Guide to offering pep | English | ✓ |
| South Sudan | 2022 | Consolidated guidelines for prevention and treatment of hiv in south sudan | English | ✓ |
| Sudan | 2021 | National guidelines on the use of antiretroviral drugs for treating and preventing hiv infection who recommendations 2021 | English |  |
| Tanzania | 2019 | National guidelines for the management of HIV and AIDS - April 2019 | English | ✓ |
| Thailand | 2020 | Thailand National Guidelines on HIV AIDS Diagnosis, Treatment and Prevention 2020 | English | Yes, but regional programs |
| Uganda | 2022 | Consolidated guidelines for the prevention and treatment of HIV and AIDS in Uganda | English | ✓ |
| Ukraine | 2019 | Новий клінічний протокол застосування антиретровірусних препаратів для лікування та профілактики ВІЛ-інфекції (A new clinical protocol for the use of antiretroviral drugs for the treatment and prevention of HIV infection) | Ukrainian | ✓ |
| Ukraine | 2022 | ВІЛ-ІНФЕКЦІЯ Клінічна настанова, заснована на доказах (HIV Infection An evidence-based clinical guideline) | Ukrainian | ✓ |
| Venezuela | 2020 | Tratamiento antir-retroviral para personas con VIH (Anti-retroviral therapy for people with HIV) | Spanish |  |
| Vietnam | 2021 | Về việc ban hành Hƣớng dẫn Điều trị và chăm sóc HIV/AIDS (On the promulgation of the Guidelines for HIV/AIDS Treatment and Care) | Vietnamese | ✓ |
| Vietnam | 2021 | Hướng dẫn quốc gia về chẩn đoán và điều trị HIV (National guidelines on HIV Diagnosis and treatment) | Vietnamese | ✓ |
| Zambia | 2022 | Zambia Consolidated Guidelines for Treatment and Prevention of HIV Infection | English | ✓ |
| Zimbabwe | 2022 | Guidelines for HIV Prevention, Testing and Treatment in Zimbabwe | English | ✓ |
| Zimbabwe | 2022 | Consolidated HIV and AIDS job aide | English | ✓ |

## Table S4 Children – 2-Drug Backbone

| **AZT/3TC** | **ABC/3TC** | **AZT or ABC (less preferred) with 3TC** | **TDF/3TC** | **TDF or ABC with 3TC** | **Others** | **No Info** |
| --- | --- | --- | --- | --- | --- | --- |
| Colombia <13y  Colombia <12y  Côte d'Ivoire  <12y <35kg  Ethiopia  India  Indonesia <10y  Nigeria  <10y <30kg  South Africa  Tanzania  Venezuela <30kg  Zambia <20kg | Cameroon  <10y <30kg  DRC <30kg  Kenya <15y <30kg  Malawi <30kg  Pakistan <20kg  South Sudan  <10y or <30kg  Sudan <10y <30kg  Uganda <30kg | Myanmar <10y  Papua New Guinea | Brazil >6y >20kg  Nigeria >30kg  South Sudan >30kg | Pakistan >20kg  Sudan >10y >30kg | Cameroon >10y >30kg TDF/FTC or 3TC  Ghana AZT / 3TC or FTC  Ukraine (AZT or ABC or TDF) / 3TC or FTC  Vietnam <10y (AZT or ABC, TDF) /3TC  Zimbabwe AZT (ABC and TDF less preferred)/3TC (FTC less preferred) | Argentina  China  Cuba  Guinea  Mexico  Moldova  Mozambique  Myanmar  Namibia  Nigeria  Russia  Rwanda  Thailand |

3TC, Lamivudine; ABC, Abacavir, AZT, Zidovudine; FTC, Emtricitabine, TDF, Tenofovir disoproxil fumarate.

## Table S5 Children – Choice of 3^rd^ Drug

| **DTG** | **DTG or** | **Protease Inhibitors** | **Others** | **No 3^rd^ drug** | **No Info** |
| --- | --- | --- | --- | --- | --- |
| Brazil >6y >20kg  DRC <30kg  India >6y >20kg  Kenya <15y <30kg  Malawi <30kg  Nigeria >30kg  Pakistan >20kg  Papua New Guinea 20-30kg  South Africa >20kg  Zimbabwe | Ghana – LPV  South Sudan >30kg – EFV  Sudan >10y >30kg – EFV  Sudan <10y <30kg – LPV/r | Colombia <13y – LPV  Colombia <12y – LPV  Côte d'Ivoire <12y <35kg – LPV  India <6y <20kg LPV or RTV  Myanmar <10y LPV or RTV  Papua New Guinea <20kg – LPV/r  South Africa <20kg – LPV  Tanzania – LPV  Venezuela <30kg – LPV/r  Zambia <20kg – LPV/r | Indonesia <10y EFV  Pakistan <20kg RAL or LPV/r  Uganda <30kg LPV/r (or DTG less preferred)  Ukraine ATV/r or DRV/r or LPV/r or RAL  Vietnam <10y DTG (LPV/r and RAL less preferred) | Cameroon >10y >30kg  Cameroon <10y <30kg  Ethiopia  Nigeria <10y <30kg  South Sudan <10y <30kg | Argentina  China  Cuba  Guinea  Mexico  Moldova  Mozambique  Myanmar  Namibia  Nigeria  Russia  Rwanda  Thailand |

ATV/R, Atazanavir/ritonavir; DRV/r, Darunavir/ritonavir; DTG, Dolutegravir; EFV. Efavirenz; LPV; Lopinavir, LPV/r, Lopinavir/ritonavir; RAL, Raltegravir; RTV, Ritonavir

## Table S6 Interventions integrated with PEP (N= 36)

| **Package of interventions prescribed with PEP** | **n/N (%) ^+^** | **Countries** |
| --- | --- | --- |
| STI testing/ prophylaxis | 27/36 (75%) | Argentina, Brazil, Cameroon, China, Colombia, Democratic Republic of the Congo, Ethiopia, Ghana, India, Indonesia, Kenya, Malawi, Mexico, Myanmar, Namibia, Nigeria, Pakistan, Papua New Guinea, Rwanda, South Africa, South Sudan, Sudan, Thailand, Uganda, Venezuela, Vietnam, Zimbabwe |
| Counselling (adherence, psychosocial support) | 27/36 (75%) | Brazil, Cameroon, China, Colombia, Democratic Republic of the Congo, Ethiopia, Ghana, India, Indonesia, Kenya, Mozambique, Myanmar, Namibia, Nigeria, Papua New Guinea, Russia, Rwanda, South Africa, South Sudan, Sudan, Tanzania, Thailand, Uganda, Ukraine, Venezuela, Vietnam, Zimbabwe |
| Emergency contraceptives | 24/36 (67%) | Argentina, Brazil, Cameroon, Colombia, Democratic Republic of the Congo, Ethiopia, Ghana, Indonesia, Kenya, Malawi, Mexico, Namibia, Nigeria, Pakistan, Papua New Guinea, Rwanda, South Africa, South Sudan, Sudan, Thailand, Uganda, Venezuela, Vietnam, Zimbabwe |
| PrEP | 18/36 (50%) | Brazil, China, Democratic Republic of the Congo, Ethiopia, Ghana, India, Indonesia, Kenya, Malawi, Mexico, Namibia, Nigeria, Pakistan, Rwanda, South Africa, South Sudan, Vietnam, Zimbabwe |
| Harm reduction | 16/36 (44%) | Brazil, Cameroon, China, Colombia, Democratic Republic of the Congo, Ghana, India, Indonesia, Kenya, Nigeria, Papua New Guinea, Rwanda, South Africa, South Sudan, Vietnam, Zimbabwe |
| Hepatitis B or hepatitis C screening and/or vaccination | 11/36 (31%) | Cameroon, Colombia, Democratic Republic of the Congo, Namibia, Pakistan, Papua New Guinea, Sudan, Tanzania, Thailand, Vietnam, Zimbabwe |
| Tetanus toxoid | 6/36 (17%) | Cameroon, Democratic Republic of the Congo, Kenya, Namibia, South Africa, Sudan |
| Criminal justice/ legal services for post-rape | 5/36 (14%) | Colombia, Ethiopia, Kenya, Namibia, Nigeria |
| Tuberculosis screening | 3/36 (8%) | Nigeria, South Africa, Zimbabwe |
| Pregnancy test only (without STI screening or emergency contraceptives) | 1/36 (3%) | Russia |
| Covid screening | 1/36 (3%) | South Africa |
| Unclear/ no information provided | 5/36 (14%) | Cote D'Ivoire, Cuba, Guinea, Moldova, Zambia |

PrEP, pre-exposure prophylaxis; STI, sexually transmitted infection
